# Improving discharge summaries from hospital with a brief recommendation text box: results from a nationwide survey

**DOI:** 10.3399/BJGPO.2024.0046

**Published:** 2024-11-27

**Authors:** Thorbjørn H Mikkelsen, Jesper B Nielsen, Maria M Storsveen, Jens Søndergaard

**Affiliations:** 1 Research Unit of General Practice, Department of Public Health, University of Southern Denmark, Odense, Denmark

**Keywords:** patient safety, general practice, surveys and questionnaires

## Abstract

**Background:**

Danish hospital physicians are obliged to mark discharge summaries addressing whether the GP is recommended to follow up the patient, as well as stating suggested follow-up actions in a recommendation text box.

**Aim:**

To investigate GPs’ experiences with the recommendation text box in discharge summaries.

**Design & setting:**

A questionnaire was sent to a representative sample of GPs in Denmark in January 2021.

**Method:**

A questionnaire was prepared for GPs based on background material, focus group interviews, and discussions with GPs and hospital physicians. It was subsequently pilot-tested by fellow researchers and GPs, and revised before the survey.

**Results:**

Seventy-two per cent of the GPs surveyed ‘totally agree' or 'partly agree’ that the recommendation text box is easy to find. In addition, our results show significant differences on how difficult the recommendation box is to find on different software. Sixty-three per cent ‘totally agree’ or ‘partly agree’ that the recommendation text box provides brief and precise information about the recommended follow-up.

**Conclusion:**

GPs generally find that the recommendation text box provides them with brief and precise information about the recommended follow-up. In addition, the software used by the GPs has a significant influence on how the recommendation text box is to find.

## How this fits in

Follow-up by the GP after discharge from hospital is important for patient safety and reduces the risk of hospital readmission. Danish hospital physicians are obliged to mark the discharge summaries addressing whether the GP is recommended to follow-up the patient as well as to suggest any follow-up action. This study shows that the Danish GPs find that the recommendation text box provides them with brief and precise information about the recommended follow-up. It also demonstrates that the software used by the GPs has a significant influence on how easy the recommendation text box is to find.

## Introduction

Follow-up by the GP after the patient’s discharge from hospital is important for patient safety and reduces the risk of hospital readmission.^1^ Despite this, GPs often have little time to review discharge summaries,^2,3^ and failures occur in the processing of requested actions in almost half of all discharge summaries.^4–7^

To improve patient safety and handover to general practice, marked discharge summaries (MDS) with a brief recommendation to the patient’s GP about how to follow-up on the patient, have been introduced in Denmark. The intention was that, by only including the necessary information in the MDS, discharge summaries would be both more focused and easier to find than they were before 2019.^8^ Since 2019, hospital physicians have been obliged to mark up the discharge summaries to signify whether the GP is recommended to follow-up on the patient as well as briefly and precisely to state the suggested follow-up action in a recommendation text box.^8,9^ Another reason for creating the recommendation text box was to make it easy to find in the discharge summary, as it is located at the beginning of the MDS.^9,10^ In this study, we give a short and focused discussion on GPs' experiences with the recommendation text box in relation to the primary aims of the box, which are that it is easy to find and that it provides brief and precise information about the recommended follow-up, as this was some of the main reasons for introducing the MDSs.

Each region in Denmark has its own electronic medical record (EMR) in which the discharge summaries are made. However, Region Zealand and the Capital Region have the same EMRs. The GPs were using seven different EMRs, each of which displays the discharge summaries in a way they find suitable. These aspects, in combination with demographic characteristics, such as the type of clinic, age, and sex, may influence the GP's perception of the new solution.

Discharge summaries have previously contained a recommendation. With the MDS these recommendations are to be placed at the top of the discharge summary by the software, stressing that it should provide brief and precise information about the recommended follow-up. The recommended follow-up is not an assignment; the GP may always, based on their knowledge of the patient, make an independent assessment of whether they find it relevant to follow the recommendation.^9^ The GP's obligations regarding the discharged patient have not been changed, the recommendation box is rather an extra service making it easy and fast to determine if and what the GP needs to be aware of after discharge from hospital. The recommendations are not followed by funding per se; however, Danish GPs are paid by a mixture of per-capita payment and fees for services. Two-thirds come from fee-for-service payments and follow-up actions are reimbursed.^10^

The guide regarding the MDS with a recommendation text box states that:^8,9^

- the hospital physician must mark the discharge summaries in which follow-up by the GP is recommended;
- the hospital physician must fill in the recommendation text box on marked MDSs; and
- within given time limits, GPs must review discharge summaries marked as containing a recommendation for follow-up on discharge.
  - Markings and follow-up: red: within 1–2 working days; yellow: within 14 days; green: before initial patient contact; no colour marking: no recommended follow-up.

To our knowledge, in 2019 Denmark became the first country to implement MDS with a recommendation text box. Therefore, it is important now to examine if the MDS is seen as supporting the work of GPs and the continuity of care.

### Aim

This study aims to investigate the GPs’ experiences with the MDSs recommendation text box.

## Method

### Study population

A questionnaire was sent to a representative sample of GPs in Denmark by the Danish Organisation of General Practitioners (PLO), the professional body for all GPs in Denmark. The questionnaire survey was carried out from 5–26 January 2021. Non-responders received a reminder on 19 January 2021. It took approximately 15 minutes to answer the questionnaire. The GPs were remunerated in accordance with instructions laid down by The Danish College of General Practitioners (DSAM), corresponding to DKK 206.43 (approximately 24 GBP) for answering the questionnaire.

The questionnaire was sent to 798 GPs.

### Questionnaire

We prepared the questionnaire for the GPs based on background material, focus group interviews, and discussions with the project advisory board, including representatives from Danish Regions, PLO, MedCom, and the Danish Patient Safety Authority. The questionnaire was constructed with answers on a 5-point Likert scale. The questionnaire was pilot-tested by fellow researchers and GPs, and revised before the survey.

### Data analysis

Our primary aim was to investigate whether the GPs experienced that the recommendation field was easy to find and if it provides brief and precise information about recommended follow-up. GPs answering, ‘totally agree’ and ‘partly agree’ to these questions are perceived to evaluate the items positively. We found that logistic regression was the best statistical method to investigate if the other listed aspects in table 3 and 4 influence whether the GPs' experiences are positive or not. Both unadjusted and adjusted analyses were carried out. We adjusted for the influence of region, EMR, sex, age group (aged ≤55 years versus aged >55 years), type of clinic (solo versus multi), and whether the clinic employs nurses. The rationale for including the list of covariates in the adjusted multivariate models is that sex and age could possibly affect the GPs’ willingness to embrace new electronic solutions. The size of the clinic and whether it employs nurses may influence how used the GPs are to taking advice from colleagues and hence how they perceive recommendations from other healthcare professionals. We applied an adjusted logistic regression, adjusting for geographic region, software used by the clinics, sex, age group, type of clinic, and whether the clinic employed one or more nurses. A *P* value of ≤0.05 was considered statistically significant. Stata (version 18) was used for analysis.

## Results

The questionnaire was sent to 798 GPs, of whom 310 responded, corresponding to a response rate of 38.8%. As can be seen from Table 1, the sample and the responders closely resemble the composition of Danish GPs. The age, sex, and geographical distribution, among those who answered, is largely similar to that among all Danish GPs and the distribution of the responses is similar to the distribution of GPs in the regions (Table 1).

**Table 1.** Characteristics of responding GPs (*n* = 310) compared with all Danish GPs

	Population of Danish GPs		Sample of GPs		Responding GPs	
	*n*	%	*n*	%	*n*	%
**Sex**						
Male	1499	43	340	43	144	46
Female	1974	57	458	57	166	54
**Geographic region**						
The Capital Region of Denmark	1064	31	248	31	88	28
The Central Denmark Region	441	13	87	11	41	13
The North Denmark Region	819	24	200	25	79	25
Region Zealand	851	25	197	25	78	25
Region of Southern Denmark	298	9	66	8	24	8
**Age, years**						
30–40	320	9	81	10	30	10
41–50	1421	41	316	40	119	38
51–60	989	28	229	29	95	31
61–70	660	19	154	19	61	20
>70	83	2	18	2	5	2

The distribution of software is approximately the same among Danish GP clinics and among the responding GPs (Table 2).

**Table 2.** The distribution of software in general practice clinics and among the GPs who have answered the questionnaire

	Distribution of software among Danish GP clinics		Distribution of software among responding GPs	
**Software used by the clinics**	*n*	%	*n*	%
MDS 1	725	42	133	43
MDS 2	436	25	87	28
MDS 3	201	12	27	9
MDS 4	239	14	46	15
MDS 5, MDS 6, MDS 7^a^	114	7	27	9
MDS 8	1	0	0	0

^a^MDS 5, MDS 6, MDS 7 are merged owing to few users.

MDS = marked discharge summaries.

Our results show that 40% ‘totally agree’ and 32% ‘partly agree’ that the recommendation text box is easy to find, while 12% ‘neither agree nor disagree’, and, respectively, 9% and 7% ‘partly disagree’ and ‘totally disagree’ (Table 3).

**Table 3.** Usability of the recommendation text box

Question	Totally agree, *n* (%)	Partly agree, *n* (%)	Neither agree nor disagree, *n* (%)	Partially disagree, *n* (%)	Totally disagree, *n* (%)	*n*
The recommendation text box is easy to find	124 (40)	98 (32)	37 (12)	29 (9)	22 (7)	310
The recommendation text box provides brief and precise information about recommended follow-up	63 (20)	134 (43)	60 (19)	39 (13)	14 (5)	310

One-fifth of the GPs (20%) ‘totally agree’ and 43% ‘partly agree’ that the recommendation text box provides brief and precise information about recommended follow-up, while nearly one-fifth (19%) ‘neither agree nor disagree’, and 13% and 5%, respectively, ‘partly disagree’ and 'totally disagree’ (Table 3).

After adjusting for geographical region, sex, age groups, type of clinic, and whether the clinic employs nurses, there were no statistical differences in the perception of whether the recommendation text box is easy to find. Our results show that the recommendation text box is significantly more difficult to find in the second-most (MDS 2, *P* value 0.014) and fourth-most (MDS 3, *P* value 0,03*3*) frequently used software compared with the most widespread software used by the GPs (MDS 1) (Table 4).

**Table 4.** The recommendation field is easy to find

	Prevalence	'Totally agree' or 'Partly agree'	'Neither agree nor disagree', 'Partially disagree', or 'Totally disagree'	OR (95% CI)^a^		Adjusted OR (95% CI)^b^	
		*n* (%)	*n* (%)		*P* value		*P* value
**Geographical region**							
The Central Denmark Region	78	58 (74.4%)	20 (25.6%)	1		1	
The Capital Region of Denmark	88	61 (69.3%)	27 (30.7%)	0.78 (0.39 to 1.54)	0.473	1.01 (0.48 to 2.14)	0.982
The North Denmark Region	24	19 (79.2%)	5 (20.8%)	1.31 (0.43 to 3.98)	0.633	1.30 (0.38 to 4.42)	0.675
Region Zealand	41	21 (51.2%)	20 (48.8%)	**0.36 (0.16 to 0.80**)*	0.012*	0.46 (0.20 to 1.04)	0.063
Region of Southern Denmark	79	63 (79.7%)	16 (20.3%)	1.36 (0.64 to 2.87)	0.424	1.42 (0.65 to 3.08)	0.378
**Software used by the clinics**							
MDS 1	133	108 (81.2%)	25 (18.8%)	1		1	
MDS 2	87	57 (65.5%)	30 (34.5%)	**0.44 (0.24 to 0.82**)*	0.010*	**0.44 (0.23 to 0.84**)*	0.014*
MDS 3	27	15 (55.6%)	12 (44.4%)	**0.29 (0.12 to 0.70**)*	0.006*	**0.34 (0.13 to 0.92**)*	0.033*
MDS 4	46	31 (67.4%)	15 (32.6%)	0.48 (0.22 to 1.02)	0.056	0.47 (0.20 to 1.09)	0.078
MDS 5, MDS 6, MDS 7^c^	17	11 (64.7%)	6 (35.3%)	0.42 (0.14 to 1.26)	0.122	0.53 (0.16 to 1.73)	0.296
**Sex**							
Male	144	96 (66.7%)	48 (33.3%)	1		1	
Female	166	126 (75.9%)	40 (24.1%)	1.57 (0.96 to 2.59)	0.073	1.52 (0.89 to 2.57)	0.124
**Age, years**							
≤55	194	148 (76.3%)	46 (23.7%)	1		1	
>55	116	74 (63.8%)	42 (36.2%)	**0.55 (0.33 to 0.91**)*	0.019*	0.59 (0.34 to 1.03)	0.062
**Type of clinic**							
Multi^d^	235	169 (71.9%)	66 (28.1%)	1		1	
Solo^e^	75	53 (70.7%)	22 (29.3%)	0.94 (0.53 to 1.67)	0.835	1.46 (0.73 to 2.92)	0.289
**One or more nurses employed in the practice?**							
Yes	275	199 (72.4%)	76 (27.6%)	1		1	
No	35	23 (65.7%)	12 (34.3%)	0.73 (0.35 to 1.55)	0.413	0.68 (0.30 to 1.55)	0.360

^a^Crude prevalence ratio is the unadjusted prevalence ratio. ^b^Adjusted logistic regression. Adjusted for geographic region, software used by the clinics, sex, age group, type of clinic and whether the clinic employs one or more nurses. ^c^MDS 5, MDS 6, MDS 7 are merged owing to few users. ^d^Partnership clinics. ^e^Solo, shared single and groups of solo GPs sharing clinic facilities.

MDS = marked discharge summaries.

Bold and asterisked = statistically significant.

There were no statistical differences after adjustment between geographical region, age groups, clinic type, or whether the clinic employ nurses in the perception of whether the recommendation text box is perceived to provide a brief and precise information about recommended follow-up (Table 5). Being female is a statistically significant predictor for agreeing that the recommendation text box provides brief and precise information about recommended follow-up, showing that female GPs answer more positively than male GPs (*P* value = 0.005) (Table 5).

**Table 5.** The recommendation text box provides brief and precise information about recommended follow-up

	Prev.	'Totally agree' or 'Partly agree'	'Neither agree nor disagree', 'Partially disagree', or 'Totally disagree'	OR (95% CI)^a^	*P* value	Adjusted OR (95% CI)^b^	*P* value
		*n* (%)	*n* (%)				
**Geographical region**							
The Central Denmark Region	78	50 (64.1%)	28 (35.9%)	1		1	
The Capital Region of Denmark	88	55 (62.5%)	33 (37.5%)	0.93 (0.50 to 1.76)	0.831	0.99 (0.50 to 1.95)	0.970
The North Denmark Region	24	14 (58.3%)	10 (41.7%)	0.78 (0.31 to 2.00)	0.610	0.75 (0.27 to 2.08)	0.577
Region Zealand	41	21 (51.2%)	20 (48.8%)	0.59 (0.27 to 1.27)	0.176	0.65 (0.30 to 1.43)	0.288
Region of Southern Denmark	79	57 (72.2%)	22 (27.8%)	1.45 (0.74 to 2.85)	0.281	1.48 (0.74 to 2.95)	0.265
**Software used by the clinics**							
MDS 1	133	91 (68.4%)	42 (31.6%)	1		1	
MDS 2	87	53 (60.9%)	34 (39.1%)	0.72 (0.41 to 1.27)	0.254	0.74 (0.41 to 1.34)	0.319
MDS 3	27	15 (55.6%)	12 (44.4%)	0.58 (0.25 to 1.34)	0.201	0.60 (0.24 to 1.52)	0.281
MDS 4	46	29 (63.0%)	17 (37.0%)	0.79 (0.39 to 1.59)	0.505	0.79 (0.36 to 1.73)	0.555
MDS 5, MDS 6, MDS 7^c^	17	9 (52.9%)	8 (47.1%)	0.52 (0.19 to 1.44)	0.209	0.56 (0.19 to 1.65)	0.292
**Sex**							
Male	144	79 (54.9%)	65 (45.1%)	1		1	
Female	166	118 (71.1%)	48 (28.9%)	**2.02 (1.26 to 3.24**)*	0.003*	**2.00 (1.23 to 3.25**)*	0.005*
**Age (years of age**)							
≤55	194	126 (64.9%)	68 (35.1%)	1		1	
>55	116	71 (61.2%)	45 (38.8%)	0.85 (0.53 to 1.37)	0.509	0.93 (0.55 to 1.57)	0.785
**Type of clinic**							
Multi^d^	235	148 (63.0%)	87 (37.0%)	1		1	
Solo^e^	75	49 (65.3%)	26 (34.7%)	1.11 (0.64 to 1.91)	0.713	1.47 (0.76 to 2.86)	0.257
**One or more nurses employed in the clinic?**							
Yes	275	175 (63.6%)	100 (36.4%)	1		1	
No	35	22 (62.9%)	13 (37.1%)	0.97 (0.47 to 2.01)	0.928	0.86 (0.39 to 1.86)	0.697

^a^Crude prevalence ratio is the unadjusted prevalence ratio. ^b^Adjusted logistic regression. Adjusted for geographic region, software used by the clinics, sex, age group, type of clinic, and whether the clinic employs one or more nurses. ^c^MDS 5, MDS 6 and MDS 7 are merged owing to few users. ^d^Partnership clinics. ^e^Solo, shared single and groups of solo GPs sharing clinic facilities.

MDS = marked discharge summaries.

Bold and asterisked = statistically significant.

## Discussion

### Summary

This study investigates GPs' experiences with the recommendation text box, aiming to determine whether or not it is easy to find and it provides brief and precise information about the recommended follow-up, as this was one of the main reasons for introducing the MDSs.^9^

This study shows that the new recommendation text box is easy to find for most GPs. GPs using the most widely used software find it relatively easy, while users of the second-most (MDS 2) and fourth-most (MDS 3) widespread software find it significantly more difficult to find it in their software. This result shows that it is important that the suppliers of software optimise their product to ensure the recommendation text box is easy to find. This is also important because our results show that almost two-thirds find that the recommendation text box provides GPs with brief and precise information about the recommended follow-up on discharge from hospital, which has been requested elsewhere.^4,7,11–13^ Hence the experiences of Danish GPs may inspire healthcare services elsewhere to improve discharge summaries with a recommendation text box at the top of the discharge summaries, thereby improving handover to general practice. In addition, our results show that statistically significantly more female than male GPs find the information brief and precise.

To our knowledge, Denmark was the first country to implement the MDSs with a recommendation text box. The results are positive, showing that the recommendation text box is easy to find and provides brief and precise information about recommended follow-up. Both aspects enhance the continuity of care and reduce information overload.

### Strengths and limitations

The purpose of the new recommendation text box was to provide brief and precise information about recommended follow-up by the patient’s GP.^9,14^ This questionnaire does not provide an unambiguous answer to this question, as we have not asked hospital physicians if they feel they have had the opportunity to provide brief and precise information about recommended follow-up. However, it is considered a strength that this study provides a valid answer from the target group of the discharge summaries, the GPs. In addition, it is also considered a strength that the sample and the responders closely resemble the composition of the wider population of Danish GPs in terms of sociodemographic characteristics and the EMR software used. The response rate of 38.8% is a limitation to this study but is not uncommon in surveys of GPs.^15–17^ This may lead to biased results, since GPs who feel the strongest about the new discharge summaries are probably those most inclined to answer the questionnaire, regardless of whether they like or dislike the changes.

Moving workload from secondary care to primary care is an ongoing issue, and is a relevant consideration regarding discharge summaries.^4^ A limitation to this study is that it does not investigate if the GPs feel that the hospitals are passing off workload.

It is a strength of this study that the guideline for filling out the text box, the software used to handle the discharge summaries, and the user interface have not changed since the data were collected, except that the software used in Region Zealand and The Capital Region has shortened the discharge summaries and removed unnecessary information.

### Comparison with existing literature

Follow-up plans have previously been identified as important for good communication between hospitals and GPs,^4,7,11–13^ and research has shown that follow-up plans are often inadequately described in discharge summaries.^4,7,13,18^ However, our results cannot show whether the recommendation text box has improved the information regarding follow-up, since we have no baseline data to compare with.

Previous research suggests that discharge summaries should contain a ‘GP action’ text box.^4^ The recommendation text box seems to provide such a ‘GP action’ box.

Region Zealand and the Capital Region use the same EMR,^19^ hence it could be expected that the results would be similar. However, in the Capital Region 69.3% 'totally agree' or 'partly agree' that the recommendation text box is easy to find, compared with the 51.2% in Region Zealand. While the differences are not statistically significant on adjustment, they do indicate that the results are being influenced by other aspects in the two regions. Since the EMR is the same, the differences might be owing to the EMR being used in different ways. Automatically generated codes, which may only be used if deemed relevant for the GP,^9^ might account for some of the differences if not used properly.^20^

### Implications for research and practice

The survey shows that the GPs find that the recommendation text box provides the GP with brief and precise information about the recommended follow-up. It also shows a significant influence of the software used by the GPs to present the MDS. In addition, it shows relatively large (yet statistically insignificant) regional differences in the GPs’ experiences of the MDS. Future studies should explore the GPs’ experiences with the new discharge letters in further detail.

## Data

The datasets are not publicly available due to regulations from The Danish Data Protection Agency.
